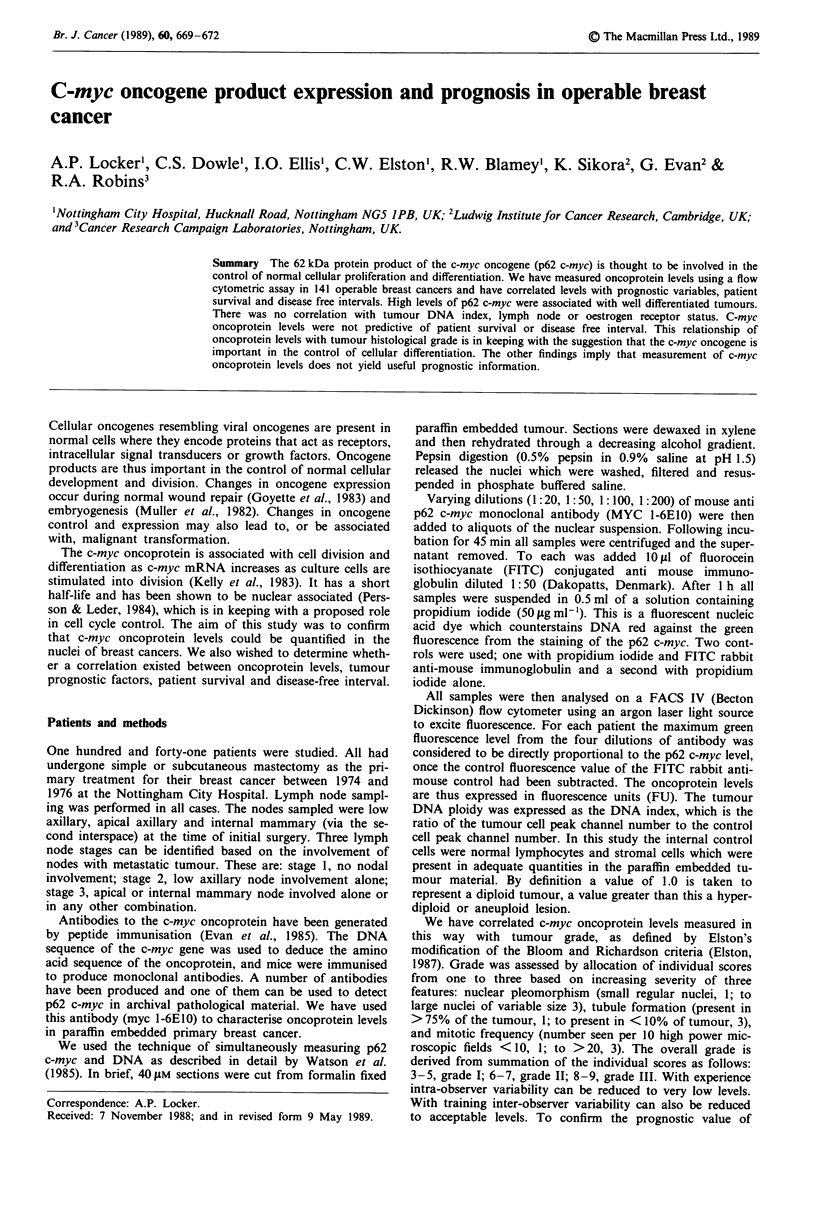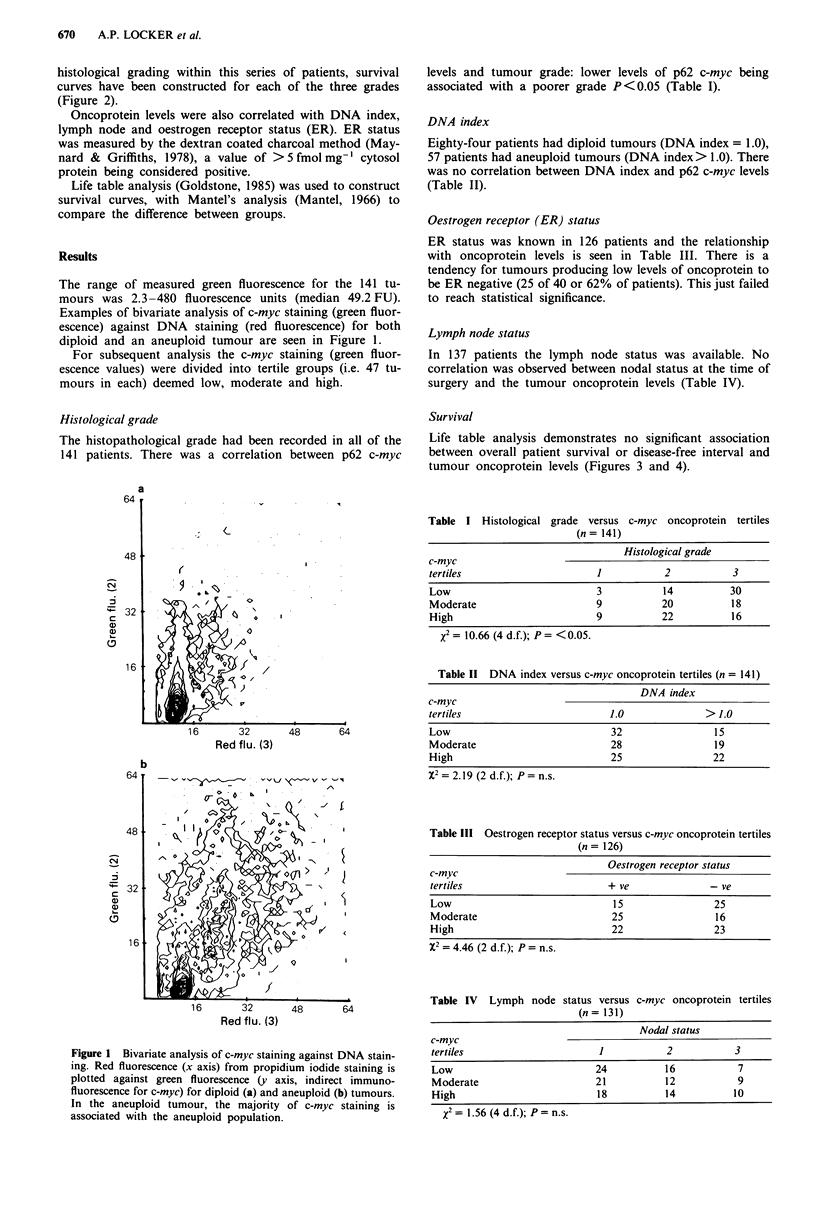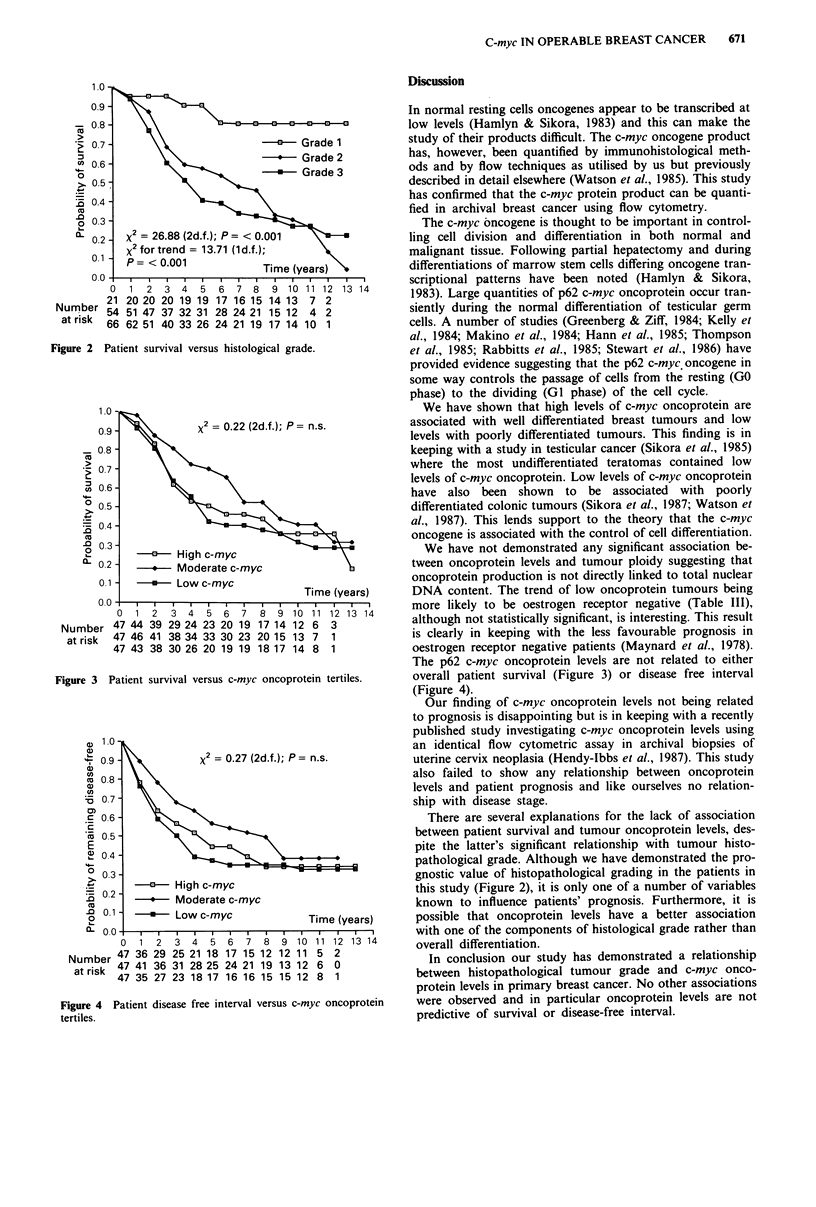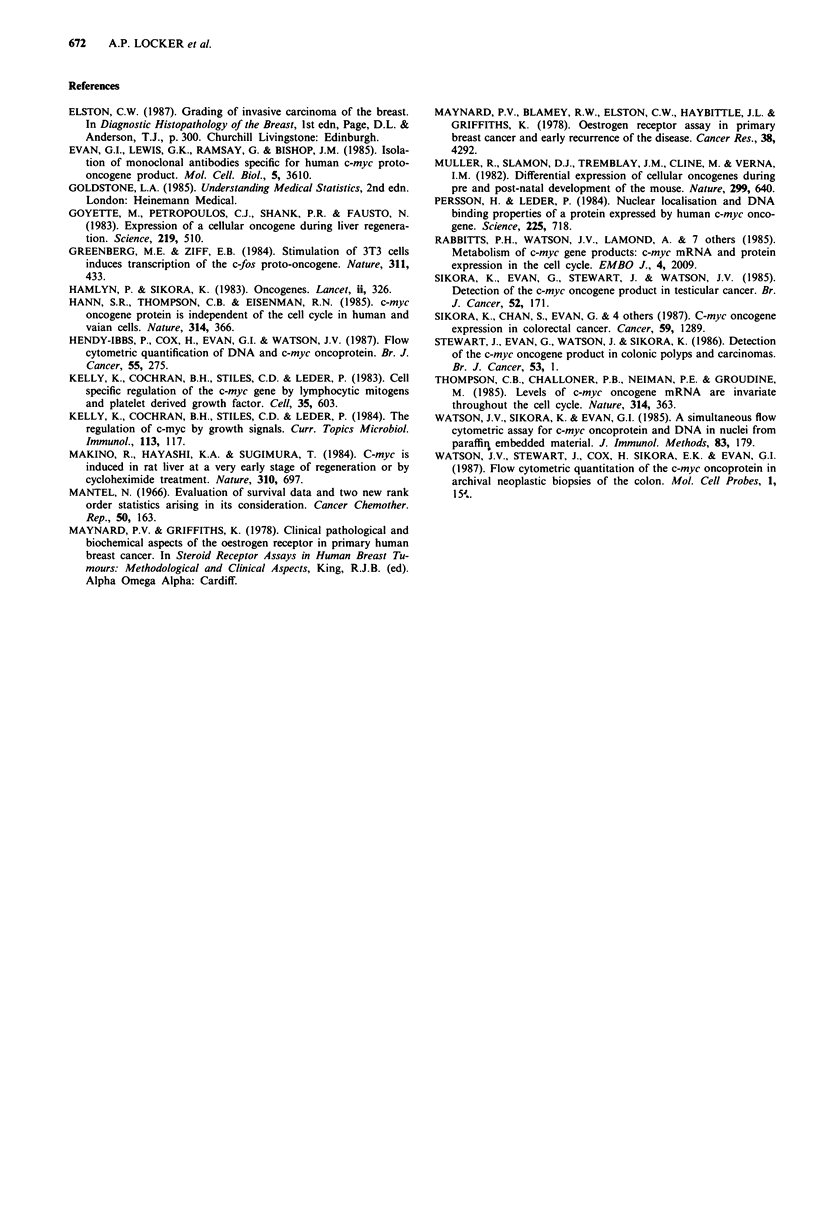# c-myc oncogene product expression and prognosis in operable breast cancer.

**DOI:** 10.1038/bjc.1989.337

**Published:** 1989-11

**Authors:** A. P. Locker, C. S. Dowle, I. O. Ellis, C. W. Elston, R. W. Blamey, K. Sikora, G. Evan, R. A. Robins

**Affiliations:** Nottingham City Hospital, UK.

## Abstract

The 62 kDa protein product of the c-myc oncogene (p62 c-myc) is thought to be involved in the control of normal cellular proliferation and differentiation. We have measured oncoprotein levels using a flow cytometric assay in 141 operable breast cancers and have correlated levels with prognostic variables, patient survival and disease free intervals. High levels of p62 c-myc were associated with well differentiated tumours. There was no correlation with tumour DNA index, lymph node or oestrogen receptor status. C-myc oncoprotein levels were not predictive of patient survival or disease free interval. This relationship of oncoprotein levels with tumour histological grade is in keeping with the suggestion that the c-myc oncogene is important in the control of cellular differentiation. The other findings imply that measurement of c-myc oncoprotein levels does not yield useful prognostic information.


Br. J. Cancer (1989), 60, 669-672 © The Macmillan Press Ltd., 1989

### C-*myc* oncogene product expression and prognosis in operable breast cancer

A.P. Locker[1], C.S. Dowle[1], I.O. Ellis[1], C.W. Elston[1], R.W. Blamey[1], K. Sikora[2], G. Evan[2] & R.A. Robins[3]

[1]*Nottingham City Hospital, Hucknall Road, Nottingham NG5 1PB, UK;* [2]*Ludwig Institute for Cancer Research, Cambridge, UK; and* [3]*Cancer Research Campaign Laboratories, Nottingham, UK.*

**Summary** The 62 kDa protein product of the c-*myc* oncogene (p62 c-*myc*) is thought to be involved in the control of normal cellular proliferation and differentiation. We have measured oncoprotein levels using a flow cytometric assay in 141 operable breast cancers and have correlated levels with prognostic variables, patient survival and disease free intervals. High levels of p62 c-*myc* were associated with well differentiated tumours. There was no correlation with tumour DNA index, lymph node or oestrogen receptor status. C-*myc* oncoprotein levels were not predictive of patient survival or disease free interval. This relationship of oncoprotein levels with tumour histological grade is in keeping with the suggestion that the c-*myc* oncogene is important in the control of cellular differentiation. The other findings imply that measurement of c-*myc* oncoprotein levels does not yield useful prognostic information.

Cellular oncogenes resembling viral oncogenes are present in normal cells where they encode proteins that act as receptors, intracellular signal transducers or growth factors. Oncogene products are thus important in the control of normal cellular development and division. Changes in oncogene expression occur during normal wound repair (Goyette *et al.*, 1983) and embryogenesis (Muller *et al.*, 1982). Changes in oncogene control and expression may also lead to, or be associated with, malignant transformation.

The c-*myc* oncoprotein is associated with cell division and differentiation as c-*myc* mRNA increases as culture cells are stimulated into division (Kelly *et al.*, 1983). It has a short half-life and has been shown to be nuclear associated (Persson & Leder, 1984), which is in keeping with a proposed role in cell cycle control. The aim of this study was to confirm that c-*myc* oncoprotein levels could be quantified in the nuclei of breast cancers. We also wished to determine whether a correlation existed between oncoprotein levels, tumour prognostic factors, patient survival and disease-free interval.

### Patients and methods

One hundred and forty-one patients were studied. All had undergone simple or subcutaneous mastectomy as the primary treatment for their breast cancer between 1974 and 1976 at the Nottingham City Hospital. Lymph node sampling was performed in all cases. The nodes sampled were low axillary, apical axillary and internal mammary (via the second interspace) at the time of initial surgery. Three lymph node stages can be identified based on the involvement of nodes with metastatic tumour. These are: stage 1, no nodal involvement; stage 2, low axillary node involvement alone; stage 3, apical or internal mammary node involved alone or in any other combination.

Antibodies to the c-*myc* oncoprotein have been generated by peptide immunisation (Evan *et al.*, 1985). The DNA sequence of the c-*myc* gene was used to deduce the amino acid sequence of the oncoprotein, and mice were immunised to produce monoclonal antibodies. A number of antibodies have been produced and one of them can be used to detect p62 c-*myc* in archival pathological material. We have used this antibody (myc 1-6E10) to characterise oncoprotein levels in paraffin embedded primary breast cancer.

We used the technique of simultaneously measuring p62 c-*myc* and DNA as described in detail by Watson *et al.* (1985). In brief, 40 $\mu$M sections were cut from formalin fixed paraffin embedded tumour. Sections were dewaxed in xylene and then rehydrated through a decreasing alcohol gradient. Pepsin digestion (0.5% pepsin in 0.9% saline at pH 1.5) released the nuclei which were washed, filtered and resuspended in phosphate buffered saline.

Varying dilutions (1:20, 1:50, 1:100, 1:200) of mouse anti p62 c-*myc* monoclonal antibody (MYC 1-6E10) were then added to aliquots of the nuclear suspension. Following incubation for 45 min all samples were centrifuged and the supernatant removed. To each was added 10 $\mu$l of fluorocein isothiocyanate (FITC) conjugated anti mouse immunoglobulin diluted 1:50 (Dakopatts, Denmark). After 1 h all samples were suspended in 0.5 ml of a solution containing propidium iodide (50 $\mu g\,ml^{-1}$). This is a fluorescent nucleic acid dye which counterstains DNA red against the green fluorescence from the staining of the p62 c-*myc*. Two controls were used; one with propidium iodide and FITC rabbit anti-mouse immunoglobulin and a second with propidium iodide alone.

All samples were then analysed on a FACS IV (Becton Dickinson) flow cytometer using an argon laser light source to excite fluorescence. For each patient the maximum green fluorescence level from the four dilutions of antibody was considered to be directly proportional to the p62 c-*myc* level, once the control fluorescence value of the FITC rabbit anti-mouse control had been subtracted. The oncoprotein levels are thus expressed in fluorescence units (FU). The tumour DNA ploidy was expressed as the DNA index, which is the ratio of the tumour cell peak channel number to the control cell peak channel number. In this study the internal control cells were normal lymphocytes and stromal cells which were present in adequate quantities in the paraffin embedded tumour material. By definition a value of 1.0 is taken to represent a diploid tumour, a value greater than this a hyperdiploid or aneuploid lesion.

We have correlated c-*myc* oncoprotein levels measured in this way with tumour grade, as defined by Elston's modification of the Bloom and Richardson criteria (Elston, 1987). Grade was assessed by allocation of individual scores from one to three based on increasing severity of three features: nuclear pleomorphism (small regular nuclei, 1; to large nuclei of variable size 3), tubule formation (present in $>75\%$ of the tumour, 1; to present in $<10\%$ of tumour, 3), and mitotic frequency (number seen per 10 high power microscopic fields $<10$, 1; to $>20$, 3). The overall grade is derived from summation of the individual scores as follows: 3–5, grade I; 6–7, grade II; 8–9, grade III. With experience intra-observer variability can be reduced to very low levels. With training inter-observer variability can also be reduced to acceptable levels. To confirm the prognostic value of

Correspondence: A.P. Locker.
Received: 7 November 1988; and in revised form 9 May 1989.

histological grading within this series of patients, survival curves have been constructed for each of the three grades (Figure 2).

Oncoprotein levels were also correlated with DNA index, lymph node and oestrogen receptor status (ER). ER status was measured by the dextran coated charcoal method (Maynard & Griffiths, 1978), a value of $>5\,\mathrm{fmol\,mg^{-1}}$ cytosol protein being considered positive.

Life table analysis (Goldstone, 1985) was used to construct survival curves, with Mantel's analysis (Mantel, 1966) to compare the difference between groups.

### Results

The range of measured green fluorescence for the 141 tumours was 2.3–480 fluorescence units (median 49.2 FU). Examples of bivariate analysis of c-*myc* staining (green fluorescence) against DNA staining (red fluorescence) for both diploid and an aneuploid tumour are seen in Figure 1.

For subsequent analysis the c-*myc* staining (green fluorescence values) were divided into tertile groups (i.e. 47 tumours in each) deemed low, moderate and high.

### *Histological grade*

The histopathological grade had been recorded in all of the 141 patients. There was a correlation between p62 c-*myc* levels and tumour grade: lower levels of p62 c-*myc* being associated with a poorer grade $P<0.05$ (Table I).

**Figure 1** Bivariate analysis of c-*myc* staining against DNA staining. Red fluorescence ($x$ axis) from propidium iodide staining is plotted against green fluorescence ($y$ axis, indirect immunofluorescence for c-*myc*) for diploid (**a**) and aneuploid (**b**) tumours. In the aneuploid tumour, the majority of c-*myc* staining is associated with the aneuploid population.

### *DNA index*

Eighty-four patients had diploid tumours (DNA index = 1.0), 57 patients had aneuploid tumours (DNA index > 1.0). There was no correlation between DNA index and p62 c-*myc* levels (Table II).

### *Oestrogen receptor (ER) status*

ER status was known in 126 patients and the relationship with oncoprotein levels is seen in Table III. There is a tendency for tumours producing low levels of oncoprotein to be ER negative (25 of 40 or 62% of patients). This just failed to reach statistical significance.

### *Lymph node status*

In 137 patients the lymph node status was available. No correlation was observed between nodal status at the time of surgery and the tumour oncoprotein levels (Table IV).

### *Survival*

Life table analysis demonstrates no significant association between overall patient survival or disease-free interval and tumour oncoprotein levels (Figures 3 and 4).

**Table I** Histological grade versus c-*myc* oncoprotein tertiles ($n = 141$)

| *c-myc tertiles* | *Histological grade* 1 | 2 | 3 |
|---|---|---|---|
| Low | 3 | 14 | 30 |
| Moderate | 9 | 20 | 18 |
| High | 9 | 22 | 16 |

$\chi^2 = 10.66$ (4 d.f.); $P = <0.05$.

**Table II** DNA index versus c-*myc* oncoprotein tertiles ($n = 141$)

| *c-myc tertiles* | *DNA index* 1.0 | >1.0 |
|---|---|---|
| Low | 32 | 15 |
| Moderate | 28 | 19 |
| High | 25 | 22 |

$\chi^2 = 2.19$ (2 d.f.); $P$ = n.s.

**Table III** Oestrogen receptor status versus c-*myc* oncoprotein tertiles ($n = 126$)

| *c-myc tertiles* | *Oestrogen receptor status* + ve | − ve |
|---|---|---|
| Low | 15 | 25 |
| Moderate | 25 | 16 |
| High | 22 | 23 |

$\chi^2 = 4.46$ (2 d.f.); $P$ = n.s.

**Table IV** Lymph node status versus c-*myc* oncoprotein tertiles ($n = 131$)

| *c-myc tertiles* | *Nodal status* 1 | 2 | 3 |
|---|---|---|---|
| Low | 24 | 16 | 7 |
| Moderate | 21 | 12 | 9 |
| High | 18 | 14 | 10 |

$\chi^2 = 1.56$ (4 d.f.); $P$ = n.s.

**Figure 2** Patient survival versus histological grade.

**Figure 3** Patient survival versus c-*myc* oncoprotein tertiles.

**Figure 4** Patient disease free interval versus c-*myc* oncoprotein tertiles.

### Discussion

In normal resting cells oncogenes appear to be transcribed at low levels (Hamlyn & Sikora, 1983) and this can make the study of their products difficult. The c-*myc* oncogene product has, however, been quantified by immunohistological methods and by flow techniques as utilised by us but previously described in detail elsewhere (Watson *et al.*, 1985). This study has confirmed that the c-*myc* protein product can be quantified in archival breast cancer using flow cytometry.

The c-*myc* oncogene is thought to be important in controlling cell division and differentiation in both normal and malignant tissue. Following partial hepatectomy and during differentiations of marrow stem cells differing oncogene transcriptional patterns have been noted (Hamlyn & Sikora, 1983). Large quantities of p62 c-*myc* oncoprotein occur transiently during the normal differentiation of testicular germ cells. A number of studies (Greenberg & Ziff, 1984; Kelly *et al.*, 1984; Makino *et al.*, 1984; Hann *et al.*, 1985; Thompson *et al.*, 1985; Rabbitts *et al.*, 1985; Stewart *et al.*, 1986) have provided evidence suggesting that the p62 c-*myc* oncogene in some way controls the passage of cells from the resting (G0 phase) to the dividing (G1 phase) of the cell cycle.

We have shown that high levels of c-*myc* oncoprotein are associated with well differentiated breast tumours and low levels with poorly differentiated tumours. This finding is in keeping with a study in testicular cancer (Sikora *et al.*, 1985) where the most undifferentiated teratomas contained low levels of c-*myc* oncoprotein. Low levels of c-*myc* oncoprotein have also been shown to be associated with poorly differentiated colonic tumours (Sikora *et al.*, 1987; Watson *et al.*, 1987). This lends support to the theory that the c-*myc* oncogene is associated with the control of cell differentiation.

We have not demonstrated any significant association between oncoprotein levels and tumour ploidy suggesting that oncoprotein production is not directly linked to total nuclear DNA content. The trend of low oncoprotein tumours being more likely to be oestrogen receptor negative (Table III), although not statistically significant, is interesting. This result is clearly in keeping with the less favourable prognosis in oestrogen receptor negative patients (Maynard *et al.*, 1978). The p62 c-*myc* oncoprotein levels are not related to either overall patient survival (Figure 3) or disease free interval (Figure 4).

Our finding of c-*myc* oncoprotein levels not being related to prognosis is disappointing but is in keeping with a recently published study investigating c-*myc* oncoprotein levels using an identical flow cytometric assay in archival biopsies of uterine cervix neoplasia (Hendy-Ibbs *et al.*, 1987). This study also failed to show any relationship between oncoprotein levels and patient prognosis and like ourselves no relationship with disease stage.

There are several explanations for the lack of association between patient survival and tumour oncoprotein levels, despite the latter's significant relationship with tumour histopathological grade. Although we have demonstrated the prognostic value of histopathological grading in the patients in this study (Figure 2), it is only one of a number of variables known to influence patients' prognosis. Furthermore, it is possible that oncoprotein levels have a better association with one of the components of histological grade rather than overall differentiation.

In conclusion our study has demonstrated a relationship between histopathological tumour grade and c-*myc* oncoprotein levels in primary breast cancer. No other associations were observed and in particular oncoprotein levels are not predictive of survival or disease-free interval.

### References

ELSTON, C.W. (1987). Grading of invasive carcinoma of the breast. In *Diagnostic Histopathology of the Breast*, 1st edn, Page, D.L. & Anderson, T.J., p. 300. Churchill Livingstone: Edinburgh.

EVAN, G.I., LEWIS, G.K., RAMSAY, G. & BISHOP, J.M. (1985). Isolation of monoclonal antibodies specific for human c-*myc* proto-oncogene product. *Mol. Cell. Biol.*, **5**, 3610.

GOLDSTONE, L.A. (1985). *Understanding Medical Statistics*, 2nd edn. London: Heinemann Medical.

GOYETTE, M., PETROPOULOS, C.J., SHANK, P.R. & FAUSTO, N. (1983). Expression of a cellular oncogene during liver regeneration. *Science*, **219**, 510.

GREENBERG, M.E. & ZIFF, E.B. (1984). Stimulation of 3T3 cells induces transcription of the c-*fos* proto-oncogene. *Nature*, **311**, 433.

HAMLYN, P. & SIKORA, K. (1983). Oncogenes. *Lancet*, **ii**, 326.

HANN, S.R., THOMPSON, C.B. & EISENMAN, R.N. (1985). c-*myc* oncogene protein is independent of the cell cycle in human and vaian cells. *Nature*, **314**, 366.

HENDY-IBBS, P., COX, H., EVAN, G.I. & WATSON, J.V. (1987). Flow cytometric quantification of DNA and c-*myc* oncoprotein. *Br. J. Cancer*, **55**, 275.

KELLY, K., COCHRAN, B.H., STILES, C.D. & LEDER, P. (1983). Cell specific regulation of the c-*myc* gene by lymphocytic mitogens and platelet derived growth factor. *Cell*, **35**, 603.

KELLY, K., COCHRAN, B.H., STILES, C.D. & LEDER, P. (1984). The regulation of c-myc by growth signals. *Curr. Topics Microbiol. Immunol.*, **113**, 117.

MAKINO, R., HAYASHI, K.A. & SUGIMURA, T. (1984). C-*myc* is induced in rat liver at a very early stage of regeneration or by cycloheximide treatment. *Nature*, **310**, 697.

MANTEL, N. (1966). Evaluation of survival data and two new rank order statistics arising in its consideration. *Cancer Chemother. Rep.*, **50**, 163.

MAYNARD, P.V. & GRIFFITHS, K. (1978). Clinical pathological and biochemical aspects of the oestrogen receptor in primary human breast cancer. In *Steroid Receptor Assays in Human Breast Tumours: Methodological and Clinical Aspects*, King, R.J.B. (ed). Alpha Omega Alpha: Cardiff.

MAYNARD, P.V., BLAMEY, R.W., ELSTON, C.W., HAYBITTLE, J.L. & GRIFFITHS, K. (1978). Oestrogen receptor assay in primary breast cancer and early recurrence of the disease. *Cancer Res.*, **38**, 4292.

MULLER, R., SLAMON, D.J., TREMBLAY, J.M., CLINE, M. & VERNA, I.M. (1982). Differential expression of cellular oncogenes during pre and post-natal development of the mouse. *Nature*, **299**, 640.

PERSSON, H. & LEDER, P. (1984). Nuclear localisation and DNA binding properties of a protein expressed by human c-*myc* oncogene. *Science*, **225**, 718.

RABBITTS, P.H., WATSON, J.V., LAMOND, A. & 7 others (1985). Metabolism of c-*myc* gene products: c-*myc* mRNA and protein expression in the cell cycle. *EMBO J.*, **4**, 2009.

SIKORA, K., EVAN, G., STEWART, J. & WATSON, J.V. (1985). Detection of the c-*myc* oncogene product in testicular cancer. *Br. J. Cancer*, **52**, 171.

SIKORA, K., CHAN, S., EVAN, G. & 4 others (1987). C-*myc* oncogene expression in colorectal cancer. *Cancer*, **59**, 1289.

STEWART, J., EVAN, G., WATSON, J. & SIKORA, K. (1986). Detection of the c-*myc* oncogene product in colonic polyps and carcinomas. *Br. J. Cancer*, **53**, 1.

THOMPSON, C.B., CHALLONER, P.B., NEIMAN, P.E. & GROUDINE, M. (1985). Levels of c-*myc* oncogene mRNA are invariate throughout the cell cycle. *Nature*, **314**, 363.

WATSON, J.V., SIKORA, K. & EVAN, G.I. (1985). A simultaneous flow cytometric assay for c-*myc* oncoprotein and DNA in nuclei from paraffin embedded material. *J. Immunol. Methods*, **83**, 179.

WATSON, J.V., STEWART, J., COX, H. SIKORA, E.K. & EVAN, G.I. (1987). Flow cytometric quantitation of the c-*myc* oncoprotein in archival neoplastic biopsies of the colon. *Mol. Cell Probes*, **1**, 151.